# Developing a Comprehensive Inventory to Define Harm Reduction Housing

**DOI:** 10.21203/rs.3.rs-4999367/v1

**Published:** 2024-10-15

**Authors:** Sofia Zaragoza, Joseph Silcox, Sabrina Rapisarda, Charlie Summers, Patricia Case, Clara To, Avik Chatterjee, Alexander Walley, Miriam Komaromy, Traci Green

**Affiliations:** The Heller School for Social Policy & Management at Brandeis University; The Heller School for Social Policy & Management at Brandeis University; The Heller School for Social Policy & Management at Brandeis University; The Heller School for Social Policy & Management at Brandeis University; Northeastern University, Bouve College of Health Sciences; Grayken Center for Addiction and Clinical Addiction Research and Education (CARE) Unit, Section of General Internal Medicine, Boston Medical Center; Grayken Center for Addiction and Clinical Addiction Research and Education (CARE) Unit, Section of General Internal Medicine, Boston Medical Center; Grayken Center for Addiction and Clinical Addiction Research and Education (CARE) Unit, Section of General Internal Medicine, Boston Medical Center; Grayken Center for Addiction and Clinical Addiction Research and Education (CARE) Unit, Section of General Internal Medicine, Boston Medical Center; The Heller School for Social Policy & Management at Brandeis University

**Keywords:** Harm Reduction, Housing, Substance Use, Qualitative Research, Outcome Measurement

## Abstract

**Background:**

The City of Boston has faced unprecedented challenges with substance use amidst changes to the illicit drug supply and increased visibility of homelessness. Among its responses, Boston developed six low threshold harm reduction housing (HRH) sites geared towards supporting the housing needs of people who use drugs (PWUD) and addressing health and safety concerns around geographically concentrated tent encampments. HRH sites are transitional supportive housing that adhere to a “housing first” approach where abstinence is not required and harm reduction services and supports are co-located. Despite the importance of HRH, the specific characteristics and operations of these sites are not well understood. This study sought to address this gap by cataloging the common features of Boston’s HRH sites to generate a comprehensive inventory tool for evaluating implementation of harm reduction strategies at transitional housing locations.

**Methods:**

We collected data between June and September 2023 and included semi-structured qualitative interviews with HRH staff (n = 19), ethnographic observations and photos at six HRH sites. Candidate inventory components were derived through triangulation of the data. Two expert medical staff unaffiliated with data collection reviewed a draft inventory measuring awareness and utility of HRH inventory components. We then pilot tested the inventory with 3 HRH residents across two sites for readability and reliability. Inventory performance was further tested in a survey of 106 residents.

**Results:**

HRH staff identified best practices, resources, and policies in HRH sites that were further contextualized with ethnographic field notes. Common to all were overdose prevention protocols, behavioral policies, security measures, and harm reduction supplies distribution. The initial 44-item inventory of services, policies and site best practices was further refined with expert and participant feedback and application, then finalized to generate a 32-item inventory. Residents identified and valued harm reduction services; medical supports were highly valued but less utilized.

**Conclusion:**

The HRH inventory comprehensively assesses harm reduction provision and residents’ awareness and perceived helpfulness of HRH operational components. Characterizing the critical components of HRH through this tool will aid in standardizing the concept and practice of HRH for PWUD and may assist other cities in planning and implementing HRH.

## BACKGROUND

Over the past several years, rates of unsheltered homelessness in the United States (U.S.) have increased significantly (1). Among people experiencing homelessness, 21% report having a serious mental illness and 16% report having a substance use disorder (2). Homelessness and housing instability put people who use drugs (PWUD) at increased risk of drug related harm, including overdose and infectious disease contraction and transmission (e.g., HIV, Hepatitis C) and especially for people who inject drugs (3, 4). Alongside these risk factors, PWUD frequently encounter heightened discrimination and barriers to accessing essential services like housing and healthcare (5, 6). Addressing risks related to substance use among unhoused populations is crucial for mitigating overdose risk and establishing housing stability.

Like many U.S. cities, Boston has faced recent unprecedented challenges with substance use amidst changes to the illicit drug supply and increased visibility of homelessness. Due to housing discrimination and their exclusion from many shelter programs, unsheltered PWUD often reside in tent encampments (7). The areas around the cross-streets of Massachusetts Avenue and Melnea Cass Boulevard in Boston, referred to as “Mass and Cass”, comprise a concentration of healthcare and social services for unhoused people that often also witnesses high rates of drug use and sales. During the COVID-19 pandemic, a tent encampment settled into this area, housing over 200 individuals at its peak occupancy (8). In response to the open-air drug market scene, frequent overdoses, and high rates of HIV transmission occurring in the encampments around Mass and Cass, the city formed six low-barrier harm reduction housing (HRH) transitional housing facilities (9, 10). The City maneuvered a mass relocation from the encampment which displaced encampment residents while also offering rapid relocation to HRH sites (11). Since their inception in January 2022, the HRH sites have housed 657 individuals, successfully moving 220 of them into permanent housing (12).

Unique to the HRH sites implemented in Boston is their explicit integration of harm reduction service connection within a Housing First model. Housing First is an evidence-based approach designed to serve unhoused individuals experiencing co-occurring substance use disorders or mental illnesses, grounded in the belief that housing is a fundamental human right (13). The original Housing First model stressed the importance of low-threshold housing without sobriety requirements, leading some authors to note the compatibility of Housing First with harm reduction approaches (14–16). Harm reduction is a philosophy that aims to reduce adverse effects of substance use in a non-judgmental, non-coercive manner, demanding that interventions reflect individual and community needs (17). Despite sharing similar core values, Housing First models often implement harm reduction only in a limited capacity, allowing individuals who are actively using substances to access housing without requiring sobriety but not implementing other widely studied harm reduction techniques (16, 18). Critics have noted a lack of explicit mention of harm reduction within broader Housing First literature (18). Implementation of harm reduction principles and services in Housing First programs is uncommon and at times has met with resistance (19). Consequently, there is little reporting on the degree to which harm reduction principles are incorporated into Housing First models, despite harm reduction’s crucial role in addressing homelessness and housing (18, 20, 21). While these programs were driven by Housing First principles, they were transitional housing, not permanent, meaning that the intention of these programs was to house people temporarily until more permanent housing settings could be found.

The goals of the Boston HRH sites were to provide both stabilization for people who had experienced living on the streets and in tent encampments, and support their transition to permanent supportive housing. The HRH models utilize a Housing First approach but are also tailored to the needs of PWUD by co-locating harm reduction services, supplies and clinical supports for residents. Thus, the foundation of the HRH model is explicit implementation of harm reduction practices and services extending beyond the absence of sobriety requirements. As this is a novel model, it remains unclear to what extent these harm reduction services and policies are present, used and perceived as helpful to residents. Our study sought to develop an inventory of HRH to identify specific characteristics and polices essential to HRH. This inventory was created with intent to be administered to staff and residents in supportive housing sites to assess the degree of harm reduction component implementation, uptake and helpfulness.

## METHODS

### Study Design & Methods

We applied a sequential design from formative to feasibility stages for inventory development. These entailed: 1) site visits and ethnographic observations; 2) semi-structured qualitative interviews with HRH program staff; 3) inventory creation and face validity assessment; and 4) pilot testing, and refinement with HRH residents.

### Site Visits and Ethnographic Observations

Beginning in June 2023, research staff conducted three ethnographic site visits across each of the HRH sites. Among the sites which permitted site visits, there were: an all-male shelter-style dormitory, an all-women space with converted office space into bedrooms, two co-ed re-purposed hotels and a tiny house community. We documented contextual evidence of site operations, structure, staffing, and location via observational field notes. When able, we took photographs of communal areas to supplement and further contextualize field notes, documenting design characteristics of each site. We took note of each HRH site’s design and operational flow. Observed elements common across HRH sites that contributed to their operations as a harm reduction space became candidate inventory items.

### HRH Staff Interviews

We invited HRH staff at each site to participate in semi-structured qualitative interviews. Our sampling design included convenient as well as purposive techniques with the intent of interviewing participants from diverse positions and with diverse expertise. Trained research staff conducted interviews using a semi-structured guide that inquired about HRH resident health and safety, experiences with service provision, and key operational aspects of their respective site. Interviews spanned between 30 and 45 minutes and were audio-recorded by research staff, then transcribed using a professional transcription service. Transcriptions were reviewed by staff and uploaded to Dedoose (22) for qualitative data analysis. HRH staff received $20 gift cards in exchange for their time and expertise.

### Inventory Creation, Face Validity Assessment

Through synthesis of prior studies on the relocated population (11, 23, 24) and data from the formative stages (25), candidate items were generated to compile initial HRH inventory checklist items for which respondents could mark components according to awareness, frequency of use, and helpfulness for staying safe from drug related harm. Once drafted, face validity checks by two medical professionals unaffiliated with the inventory construction but involved in HRH design with over 50 collective years of clinical care experience provided feedback on the inventory items and response. Providers were asked to assess the inventory components for accuracy, completeness, and fidelity to the concepts of HRH as practiced. Most items remained the same with minor refinements after the validity check.

### Pilot Testing and Refinement

We piloted the draft inventory with three HRH residents to check its readability, clarity in instructions and administration, and to elicit their feedback on the items and overall measure. Residents were compensated with $20 gift cards. The final refinement of the HRH inventory occurred after administration of the measure to 106 residents as part of the baseline data collection for the study. Residents were recruited from each of the HRH sites and invited to take part in a cross-sectional survey about their relocation to HRH sites and its effect on their health. Consenting participants were compensated $40 cash for completion of the survey, which contained the HRH inventory.

### Data Analysis

#### Qualitative data

Interview transcripts were imported into Dedoose, coded, and then analyzed using inductive and deductive approaches. A codebook developed by the research team highlighted HRH components from interviews and ethnographic field notes. Throughout the analysis process, new inductive codes were integrated into the codebook to capture emerging thematic areas beyond deductive codes derived from the interview guide and initial codebook. Codes were established by the research team to encompass various aspects of HRH (e.g., harm reduction supplies, overdose prevention strategies, co-located medical services) with items reflecting site-specific policies and services categorized as subcodes (e.g., HRH supplies: accessibility, sterile syringes, safer smoking materials, naloxone; overdose prevention strategies: room checks, safe consumption spaces, bathroom alerts, and more). Selected subcodes, reflecting the items within each category, were utilized to formulate a preliminary inventory that defined harm reduction housing. Data across interviews and ethnographic observation were then synthesized and abstracted into larger categories that became core elements of HRH and informed the drafting of inventory items.

#### Survey Inventory Data

We calculated descriptive statistics (percent of response option; median and interquartile range (25% and 75%) on the administered inventory responses to observe performance of the inventory and its ability to measure the range of experiences among HRH residents.

## RESULTS

In our results we highlight each stage of data collection that contributed to HRH inventory creation that refined the measure’s development.

### Ethnographic Observations

From June 2023 to September 2023, ethnographic site visits at five locations that permitted observation documented common aspects related to building infrastructure, on site staff, and resident security. Upon entering HRH sites there was some level of physical security present, ranging from metal detectors, security guards, and visible security cameras. While each site differed in the elaborateness of their security makeup, the intention of security and safety was consistent, thus all observed security components were catalogued.

### Front Desk Security Stations:

Observing the internal infrastructure of HRH sites, some variation of a community room or space was consistently present. The extent to which these community spaces were populated by residents during site visits depended on the time of day, with meal time hours (lunch, dinner) being most utilized.

### Common Areas:

Another observable aspect of the building infrastructure was lockers present in communal spaces. While most observed lockers appeared to be used for storage of personal belongings, some sites had lockers or lock boxes. The contents of the lockers were private and could therefore be safe places where residents could store drugs or drug use materials. The on-site lockers appeared to promote individual privacy and security. As privacy is a commodity often not afforded in traditional emergency shelters, the provision of lockers on-site was notable and therefore catalogued as an inventory item.

### Lockers:

Observations of the HRH sites also identified designated smoking areas as a distinctive feature present across all HRH locations. These smoking areas were documented by research staff as being frequently used by residents during all site visits. Research staff also noted that both illicit substances, such as opioids, crack cocaine, and methamphetamine, as well as legal substances such as tobacco and cannabis were used in these areas.

### Designated Smoking Area:

Often observed on HRH sites were medical personnel, either as directly embedded site staff or as outsourced care providers. For instance, we found that one HRH site had a nurse’s station stocked with medical supplies, including wound care kits, medications, and staffed by an on-site nurse, while another site utilized outsourced services provided by emergency medical technicians (EMTs). Occasionally during site visits we observed medical staff who were scheduled for one or two days per week care provision on-site. On site nurse stations also supplied and distributed safer drug use supplies such as sterile syringes.

### Medical Supplies in Nurse Station:

#### Harm Reduction Housing Staff Interviews

Nineteen staff from all six sites participated in interviews including three on-site clinical staff, six case workers, seven program directors, one recovery coach, one harm reduction specialist, and one resident assistant. Staff spoke to on-site HRH operations, and highlighted a commitment to harm reduction services and accessibility for residents. During site visits and interviews, staff communicated practices they perceived as essential to supporting the well-being of residents were catalogued and considered as candidate inventory items.

Across all sites, staff underscored the provision of harm reduction supplies on-site. Staff often detailed the specific harm reduction supplies they offered to residents:

“All of our guests can pick up a kit, either from our counselors or downstairs when they would like to use and go outside. So, it has syringes, cookers, alcohol wipes, all of those, you know, a safe use kit. Just consistently low barriers and expectations to try and meet our guys where they’re at.” - Site 6

Recognizing the prevalence of ongoing drug use among residents, ensuring access to safe drug use supplies emerged as another operational component, therefore the common supplies offered to residents were catalogued. Additional services that were documented as potential inventory items included medical services. While medical staff and stations were observed during field visits, staff were able to describe their specific role and the range of other medical services they provided to the residents. An on-site nurse described her role within the HRH site:

“My first thing is just making sure everybody’s breathing, they’re alive. And then beyond that, just trying to go a little bit further into care, addressing any wounds, abscesses, infections, be monitoring for that, to look for any signs of people starting to get sick, any psychological decompensation, just on trying to keep track of everybody’s health really.” - Site 3

While on-site medical staff were not available 24 hours a day, 7 days a week across all sites, site staff felt their regular presence was beneficial to access non-stigmatizing care, particularly for residents who may have encountered stigma in traditional medical settings. Thus, on-site medical care was included as a component in the inventory. The provision of medical services facilitated access not only to treatment with medications for opioid use disorder (MOUD) but also to HIV testing, prevention, and care, a critical need given the heightened HIV risk among people who inject drugs and the current Boston area HIV outbreak (26). Wound care was an additional service that many sites offered to address drug related wounds and other skin infections common among residents. Staff reported their understanding of resident preferences for wound care offered on-site as superior to accessing care in other settings, since the care offered in HRH included sterile supplies and care by trained medical personnel, but did not include the stigmatization that they reported experiencing in traditional medical settings.

“They’re more willing to engage in medical treatment, whatever that is, sometimes abscesses, it’s really hard to get anyone to go to the hospital. Since [specialized healthcare agency] comes here. some clients are more willing to engage. Like they’re like, wow, I’ve never had this much help…. So sometimes when they see things are moving for them and they actually have tangible results, they’re more willing to engage with us.” - Site 1

Harm reduction principles were integrated into the sites through provision of supplies and services as well as in their policies. While substance use policies varied across locations, all sites were committed to flexible tolerance of use. The program director of one HRH site explained how residents were not reprimanded for using substances within the HRH residence

“So, we were very flexible, and again just had supportive guiding conversations with people if we found people with drugs on them, we would talk with them. We would say ‘You need to put that in your lock box. Let’s go do that. Do you need to go out and use right now? We’ll take you down so that you can go out.’ So basically, just redirecting so that it was manageable.” - Site 5

Tolerance of substance use represented a level of flexibility and autonomy that was reflected in other on-site policies described by staff. Policies that promoted flexibility in residency, such as extended leave with the assurance of retaining one’s bed upon return, similarly underscore an elevated support for autonomy that was afforded to residents in the HRH environment compared with traditional emergency shelters that do not guarantee beds to all. Site rules, which were added to the inventory based on observation and interview data, included policies on absence and permitting cohabitation by couples. These policies, which emphasize autonomy, promote harm reduction philosophy by encouraging resident independence and choice.

Another way that staff described creating empowering and flexible environments was through facilitation of a supportive network of peers and staff. All sites, for example, incorporated scheduled community meetings for both staff and residents to attend – often consisting of updates on new policies, procedures, as well as giving residents opportunities to voice recommendations or grievances. Some sites hosted community activities that involved resident gatherings, such as pizza parties or waffle making, or recovery-oriented community events such as Alcohol Anonymous or Narcotics Anonymous meetings. Such operations were included as potential inventory items that promoted a harm reduction-oriented space of community support. Staff were able to describe how they promoted an HRH community:

“I think we really have established a community. We do things to bring the guests and the staff closer, I will always use the example of we have barbecues …, and it’s just a very informal way to engage with people. And you know, it’s a suggestion that one of our housing guests had that like I have not had a homemade burger in like 15 years like, let’s do, I want a barbecue. So, we started doing that and that was really popular and that became like a weekly thing.” – Site 1

Staff also talked about ways in which security staff contributed to this environment. While security guards were observed during site visits, staff spoke to the ways in which security guards prioritized physical safety through a person-centered approach, de-escalation, and trauma-informed actions. This approach fostered good relations with residents, emphasizing harm reduction and mutual respect, therefore it was included as a potential inventory component. A staff member from an all-women’s shelter described their security staffing:

“We have security 24/7. We require that it’s a female security staff in case they do need to intervene and use physical touch. But that doesn’t really happen. We try to avoid that at all costs.” – Site 5

The harm reduction adaptations of the existing shelter system and focused, co-located attention to basic needs (i.e., food, housekeeping) defined the HRH spaces as exceptions to a larger housing system’s typical service structure and a transitional housing service. Transitional supports such as providing daily meals and transportation proved to be integral for the well-being of residents and allowed for increased engagement with other services. One HRH case manager spoke to the importance and changes which s/he observed within residents after attending to their social determinants of health:

“It is really awesome to see people coming in off the streets and just always trying to survive, a little bit of that is taken off their shoulders and they can kind of settle in and their personalities start to come out a little more because you’re not defending for your life all the time. Because you have housing, you have stability you have food. And to see them move on and be housed is really incredible.” - Site 1

The intertwining of basic needs and harm reduction service supports present at HRH sites suggested their inclusion into the inventory as possible components.

### HRH Model Core Principles

Synthesizing across data sources and themes, six core principles of the HRH model were identified: Harm reduction supply accessibility, co-located medical services, resident autonomy and privacy, resident safety and security, community promotion, and transitional supports (Table 1).

Many of the domains outlined in Table 1 assisted in transitioning residents to permanent housing by providing not only care and safety but stability and meeting basic living needs. It is important to note that these components were not necessarily incorporated into the permanent housing to which residents were being transitioned. Additionally, not all HRH sites incorporated each inventory component uniformly and some programs did learn and adapt to include more components over time. Supplementary Table 2 includes a cross-sectional site-by-site cataloguing of HRH components.

### Pilot Testing and Refinement

Once inventory items were drafted, the response options and format were developed, with the aim of being able to assess uptake and efficacy of services according to resident experiences and staff impressions. Three scale categories were added including: 1) whether a resident was aware of the availability of the service or policy at the site; 2) how frequently the resident utilized the service; and 3) how helpful they thought the service was either in general or in regards to keeping safe including from overdose or other drug related harms. Awareness was measured on a 0 and 1 scale; 0 indicating not aware of the service, 1 indicating awareness of the service; frequency and helpfulness were initially measured on a 10-point Likert scale.

Three participants (all males with an average age of 40, residents of two sites) completed the pilot inventory and provided feedback. Residents suggested a 5-point Likert scale for assessing frequency and helpfulness, because the larger range to 10 was difficult to conceptualize. Also, staying safe from drug related harm was encouraged to be the central aspect of helpfulness that the scale should measure. For some inventory items we improved wording or included extra definitions to make clear what was being asked, as some resident had trouble understanding the specific policy to which we were referring (ex. “absence policy” was rephrased to “how long can you leave before you lose your bed?”). Items were also separated into services and policies, such that questions about frequency of use would be meaningful (e.g., metal detectors and security cameras). Items where frequency was not logical to inquire about were moved to the end and only awareness and helpfulness of these were assessed.

### Final Inventory Distribution

The final draft of the inventory was then fielded to 106 HRH residents to assess its ease of administration, whether it generated data reflective of HRH service implementation and uptake, and if the inventory detected variability in the dimension of component helpfulness. The measure proved easy to administer and feasible to complete by a trained interviewer in about 10 minutes. HRH residents from 7 sites were administered the survey tool containing the inventory. Table 2 reports the participant demographics and Table 3 reports the responses to the inventory components.

The HRH inventory generated varied responses from residents across awareness, frequency, and helpfulness (Table 3), providing insights into the uptake and utility of HRH components. Most inventory components garnered high levels of awareness across the sample, especially those related to basic needs like meals and housekeeping, but also components like harm reduction supplies and supportive policies. Awareness was lower for medical care, treatment availability, dispensed medication delivery, availability of HIV PrEP prescribing, and mental healthcare availability on site. Even if aware of the service or policy component, a number were under-utilized (i.e., less than half indicating at least some frequency). Less frequently used services or polices tended to be related to medical care, especially addiction medication treatment and HIV PrEP. There was also lower (less than 50%) awareness and therefore lower use of visitor policies, employment opportunities, peer recovery coach supports, recovery events, space dedicated to substance use consumption, and policies related to tolerance for use in their room. Most other known components were used frequently by residents. All services that were used at least some of the time and all policies known to residents were deemed very or extremely helpful (median scores of 4 or 5), except the policy of placing security cameras on site (median score 3). Presence of metal detectors and security guards on site were rated as more helpful to residents than security cameras in staying safe. From this initial application of the HRH inventory with residents, and given the lower helpfulness scores, the component relating to security cameras was removed from the final version of the 32-item HRH inventory (see Appendix, Table 1). All other components and ratings were retained. An HRH inventory adapted for administering to staff was also created but has not yet been pilot tested for feasibility (available upon request from the senior author).

## DISCUSSION

This study is the first to catalog components of harm reduction housing from observation and staff perspectives, and the first to develop an inventory characterizing key aspects of HRH that can be administered to residents. We theorized six core principles of the HRH model: 1) harm reduction supply accessibility, 2) co-located medical services, 3) resident autonomy and privacy, 4) community promotion, 5) resident safety and security, and 6) transitional support. These principles build on established Housing First philosophy (14) with an explicit harm reduction focus that reflects intentions to support PWUD in a way that traditional shelters and many housing first programs have not consistently sustained (18, 27, 28).

The HRH inventory that identifies the core tenants of the HRH model was used to develop a measure of harm reduction services and policies to catalog their provision and use in HRH sites for studying continuity, uptake, and evolution of care. The measure was feasible for replicable administration to HRH residents across all sites. The elements captured in the comprehensive inventory are important to assess among residents in an ongoing fashion, because these models are new and understudied and may need to be adjusted over time to maintain their utility to resident, staff, and community goals for housing, health, and safety. While the purpose of the inventory creation was to identify commonalities to define the HRH model, the variations in HRH operations in practice that we tabulated (see Supplementary Table 2) suggest a need for more consistent harm reduction practices implementation and longitudinal research on resident impact, efficacy and utilization of services. As these models are new, their sustainability remains unknown. More research is needed on fiscal sustainability of HRH sites, such as looking into cost saving alternatives to more expensive site operations (29). However, this inventory can be used to asses harm reduction practices implementation as well as resident utilization of services, impact, and efficacy. Future programming for shelters, supportive permanent housing or transitional housing may also use this inventory to plan, train staff, and evaluate the fidelity to harm reduction implementation within their programs.

The principles outlined may encourage new programming and provide support for the formal integration of harm reduction services into Housing First and other housing programs. Harm reduction services, such as naloxone provision and safe use supplies, have extensive evidence bases, but also require support at the staff and leadership levels for successful implementation and sustainability (30–33). Current Housing First principles should work to integrate HRH principles and practice to uniformly support PWUD.

Our research also contributes to the new and growing literature on HRH outcomes and experiences. Results from inventory administration found that residents were highly aware of basic needs services like meals and housekeeping, as well as harm reduction supplies and supportive policies. However, awareness and utilization were lower for medical care services, addiction medication treatments, HIV, PrEP, and other supportive policies. Frequently used services were often deemed helpful and meaningful by the residents. Despite its novelty, evidence is accumulating on the experiences of PWUD in HRH, particularly its effectiveness for those exiting encampments. Research with residents suggests that HRH improves both their overall health and safety, by emphasizing the importance of protecting autonomy, privacy, and healthcare access in transitional housing settings (11, 24, 34), all of which are reflected in our inventory measure.

The HRH models highlight a progressive shift in supporting PWUD experiencing homelessness, as they offer alternative solutions to addressing homelessness and encampments. Responses to the public health crisis occurring in US cities like Boston have often involved large-scale, police-led clearings through the execution of sweeps and threats of arrest. Research has shown that these types of clearings that promote mass displacement of people experiencing homelessness have negative health consequences (35, 36), but are expected actions by cities without massive investments in housing alternatives. Thus, policy and practice implementations of rapid actions include the need to center harm reduction strategies and swiftly establish housing structures and shelter adaptations that embrace HRH models. The inventory developed in this study can help standardize the planning and fidelity to HRH models, and can be used to better understand the needs of residents. The clear uptake and utility harm reduction supplies, services, and policies within transitional housing calls for future application of our HRH inventory, potentially with adaptation, to permanent support housing settings.

The findings in this study should be considered in the context of its limitations. The inventory constructed was based both on ethnographic observations and staff knowledge and experiences, with resident involvement only in later stages of the formative process. The final 32-item measure is lengthy; future efforts could consider further reduction in items. This inventory may or may not be adaptable to permanent housing locations as they don’t have the same supportive mechanisms or resources in place. Due to privacy concerns, staff were not permitted to make ethnographic observations at one of the six sites. Despite the fact that the HRH sites were intended to be transitional and temporary, several sites have unexpectedly shut down due to funding, thus the temporary nature of the HRH limited consistency of data collection. As a result, we were unable to assess resident perceptions at one of the sites. Finally, the inventory was not tested formally for test-retest reliability or content validity so future investigations should explore these characteristics and the inventory’s responsiveness to change.

## CONCLUSION

Our findings indicate that HRH sites shared common practices and policies which could be measured via a 32-item inventory. The HRH inventory is a promising tool for gauging adherence to fundamental HRH concepts and ensuring residents’ access to critical on-site HRH policies and resources. As emerging literature explores the efficacy of the HRH model, the HRH inventory can serve as a metric of model fidelity establishing parity across sites, and gauging the range of harm reduction principles being adopted by traditional shelter structures and other transitional housing settings.

## Figures and Tables

**Figure 1:**
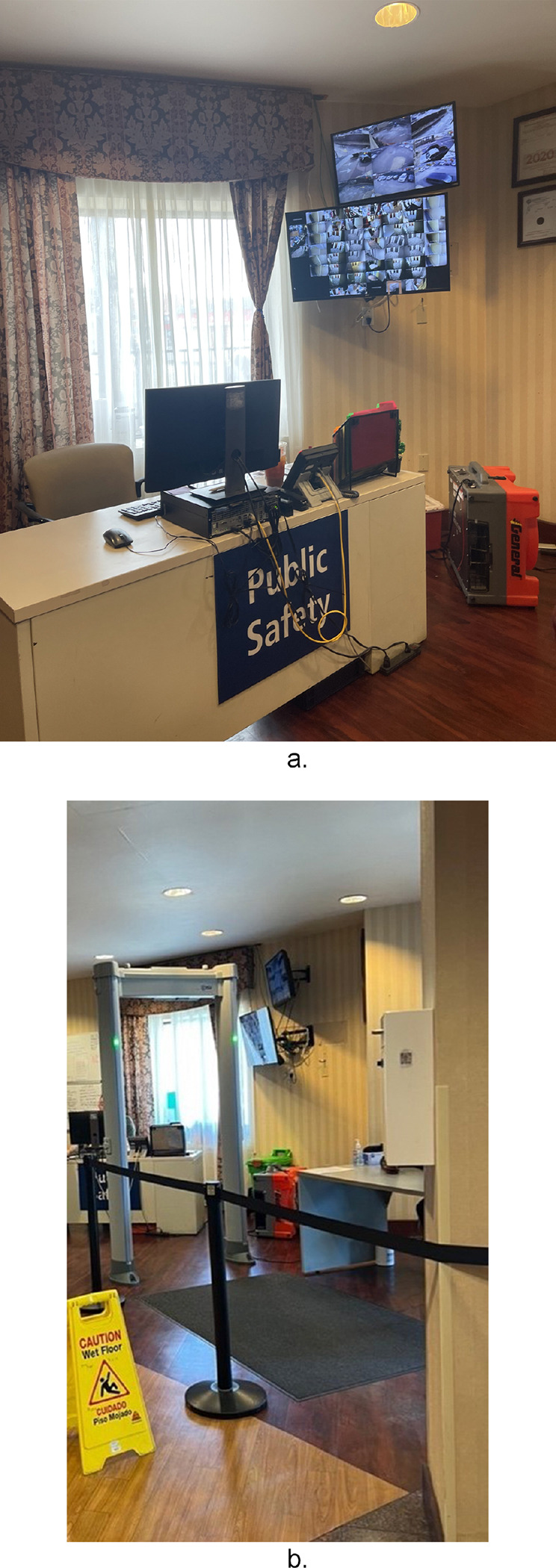


**Figure 2:**
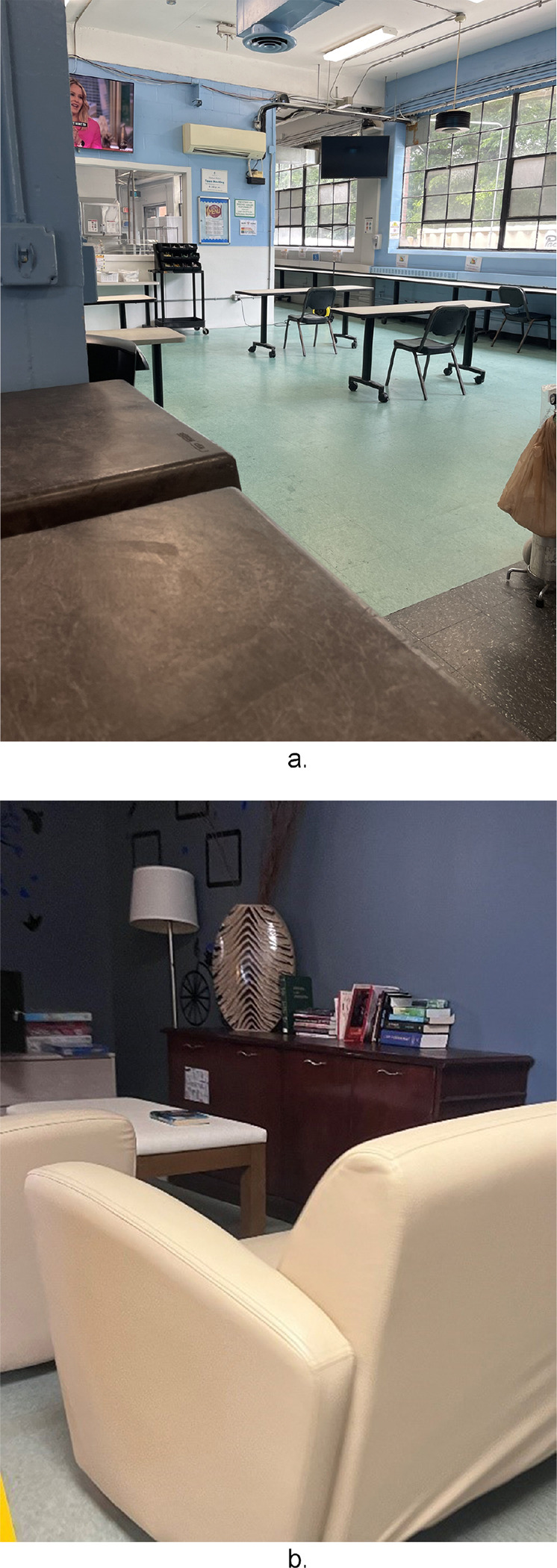


**Figure 3:**
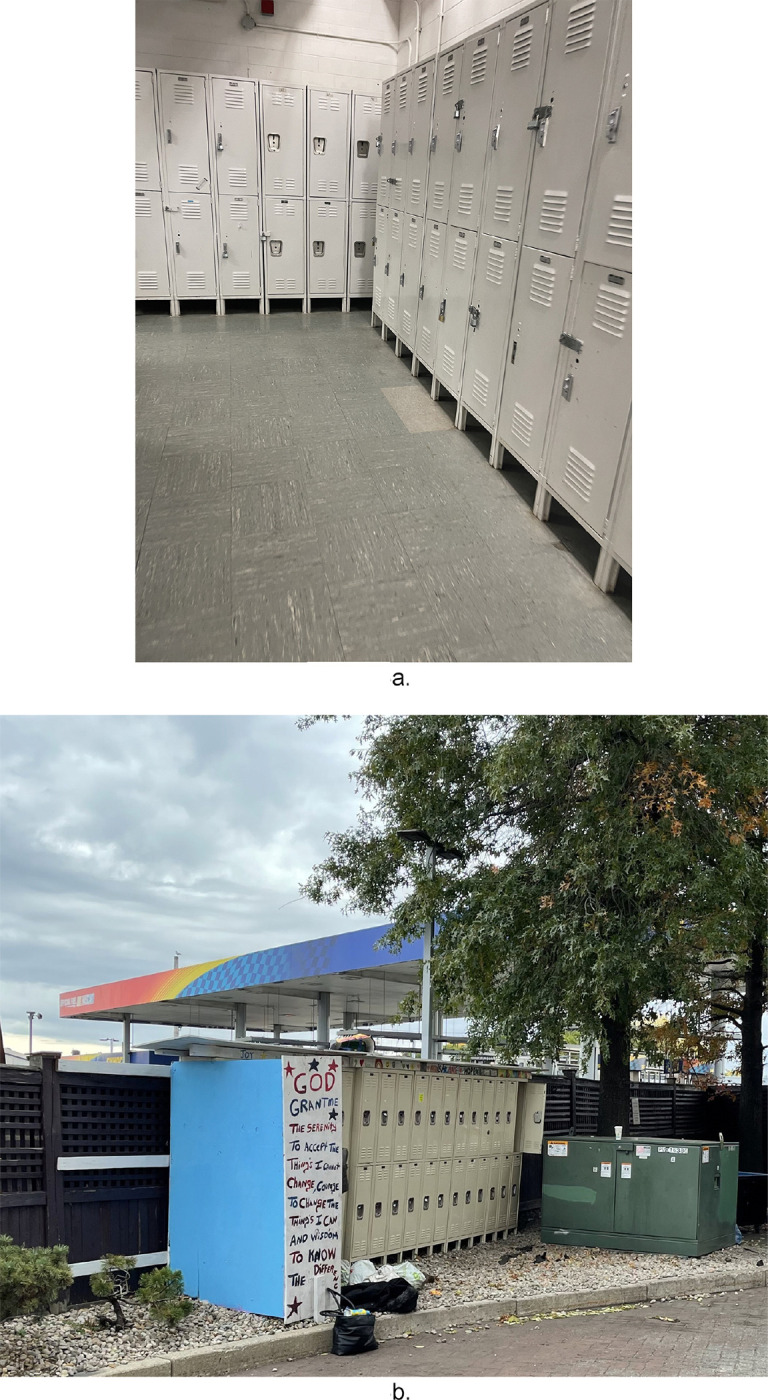


**Figure 4:**
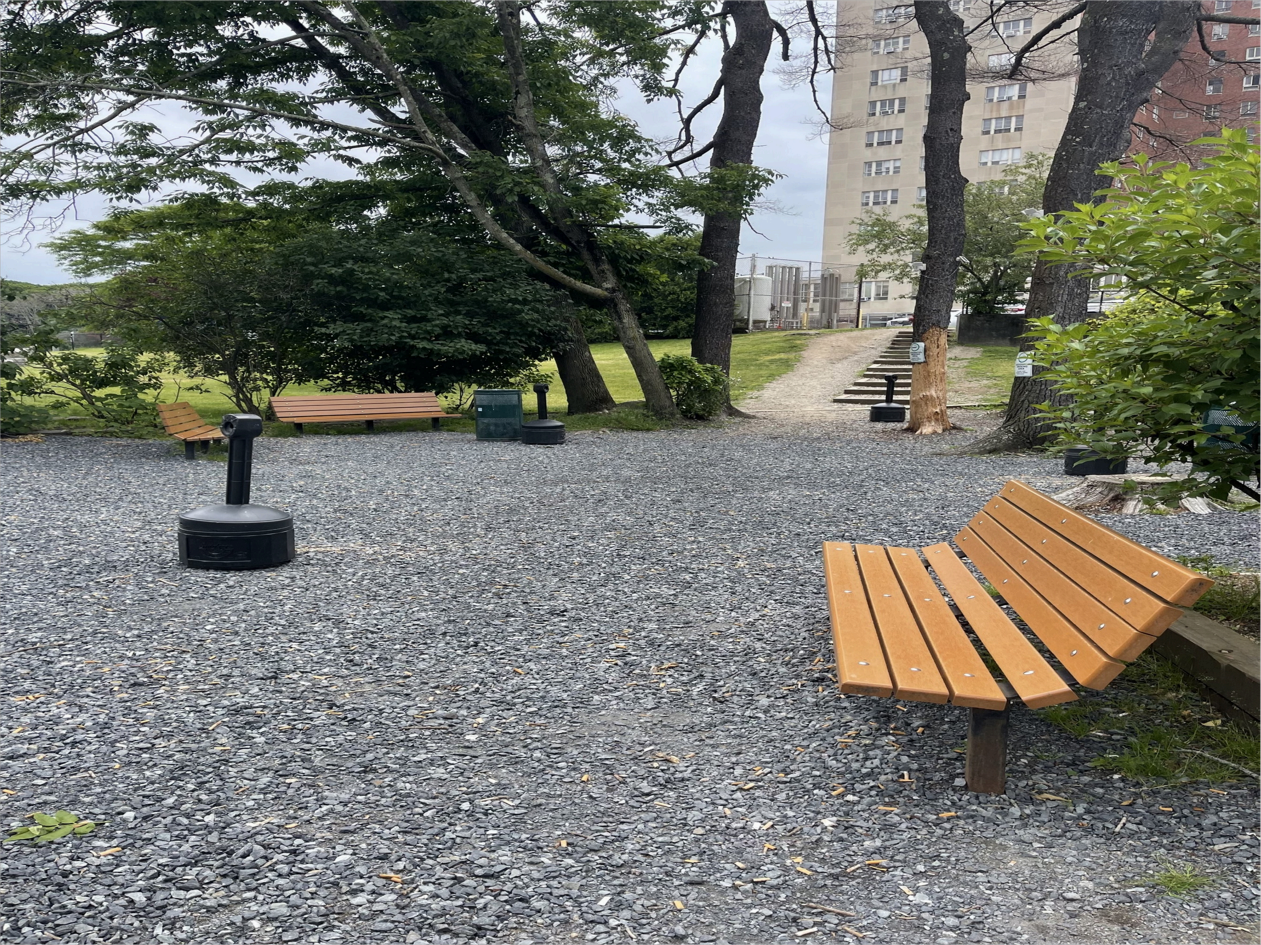


**Figure 5:**
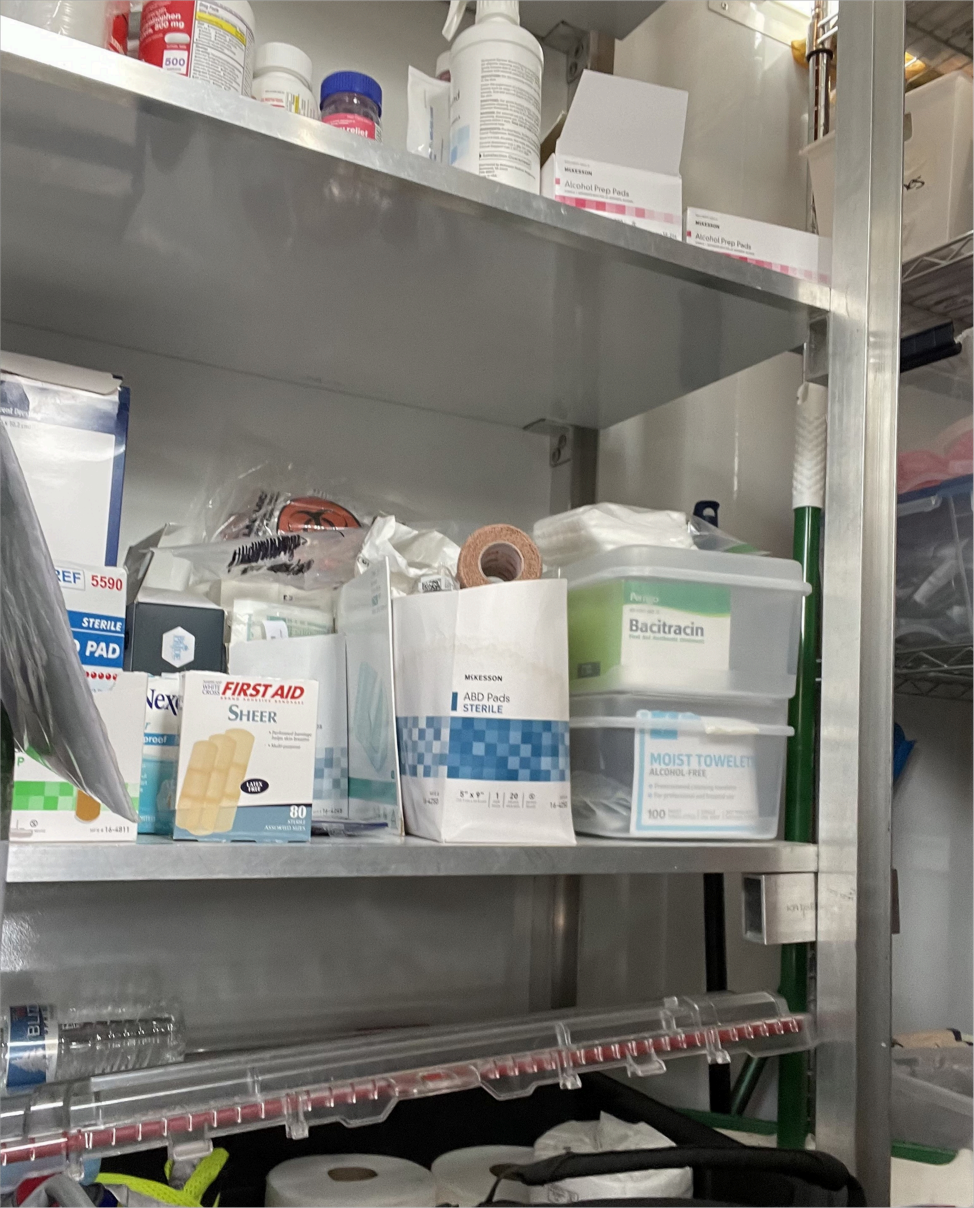


**Table 1:** Core Harm Reduction Housing Model Principles

Harm Reduction Housing^z,2^ Model Principle	Description	Corresponding Inventory Items
**Harm Reduction Supply Accessibility**	Sites strive to provide consistent access to a variety of harm reduction supplies to residents on-site.	• Harm reduction supplies available 24/7 • Sterile syringes • Smoking materials • Naloxone accessibility • Harm reduction staff training
**Co-Located Medical Services**	Sites are positioned so that key medical services are either on-site or easily accessible (including Medication for Opioid Use Disorder).	• Medication for opioid use disorder^y,3^ provided on site or nearby location • Nurse or medical staff on site 24/7 • Wound care services and supplies • HIV testing provided on site • HIV prevention and treatment medication available • Medication delivery • Mental health care on site
**Resident Autonomy and Privacy**	Sites establish policies which maximize resident autonomy, flexibility and privacy, including relating to substance use.	• Absence Policy^z,4^ ^z,2^ HRH: Harm Reduction Housing ^y,3^ MOUD: Medications for Opioid Use Disorder ^x,4^ Absence Policy allows residents to leave for extended periods of time without losing their bed (typically 7 days maximum) ^w,5^ Prosocial and pro-couple policies include ability to live with your partner or friend • Prosocial polices / Pro-couple policies^w,5^ • Lockers • Substance use tolerance on site • Substance use tolerance in rooms
**Resident Safety and Security**	Sites balance flexibility and understanding with attention to physical safety and minimization of drug-related harm.	• Women centered and gender aware services • Security guards • Metal detectors • Wellness checks/Room checks • Staff has naloxone on person at all times • Dedicated space for smoking or consuming substances • Behavioral policies
**Community Promotion**	Sites seek to encourage the development of supportive staff and peer communities.	• Recovery groups • Peer recovery coaches • Community meetings • Community room / Common space
**Transitional Supports**	Sites provide basic needs to residents as a transitional housing support.	• Housekeeping • Daily Meals • Transportation Support • Case Management

**Table 2:** Self-Reported Harm Reduction Housing Resident Demographics, N = 106

Variable	n	%
**Gender**		
Male	61	57.5
Female	43	40.5
Transgender/nonbinary	1	0.9
Other	1	0.9
**Age**		
18–25	1	0.9
26–30	6	5.7
31–35	10	9.4
36–40	25	23.6
41–45	17	16.0
46–55	30	28.3
56+	17	16.0
**Race** ^1^		
White	55	51.9
Black	29	27.4
American Indian	11	10.4
Some other race	24	22.6
**Ethnicity**		
Hispanic	30	28.3
Non-Hispanic	75	70.8
Unknown	1	0.9
**Current Drug Use** ^1^		
Cocaine	58	54.7
Crack	60	56.6
Heroin	79	74.5
Fentanyl	60	56.6
Methamphetamine	27	25.5
Marijuana	44	41.5
Other drugs^2^	39	36.8
**Current Injection Drug Use**		
Yes	65	61.3
No	39	36.8
Unknown	2	1.9
**HIV Status**		
+	8	7.5
−	96	90.6
Unknown	2	1.9

1 People could indicate more than one response.

2 ‘Other drugs’ include real or fake prescription medications and synthetic cannabinoids or were not specified.

**Table 3:** Harm Reduction Housing Inventory responses for 106 residents, Boston 2023–2024

	Aware of availability N (%)	At least some use of component (> 0 frequency) N (%)	Rating of helpfulness in staying safe, including from overdose and other drug-related harm [Median, Interquartile Range (IQR), scale 1–5]
*Available services or supplies at the harm reduction housing location*			
Access to medication for substance use disorder^z,2^ on site (buprenorphine, methadone, naltrexone, antabuse)	32 (30.2)	11 (34.4)	5 [3,5]
Access to HIV^y,3^ testing on site	53 (50)	33 (62.3)	5 [4,5]
Access to HIV medication for prevention and treatment (e.g PrEP^x,4^, antiretrovirals)	37 (34.9)	9 (24.3)	5 [4,5]
Medications are delivered to residents directly on site	49 (46.2)	32 (65.3)	5 [5,5]
A nurse or medical staff on site 24/7	62 (58.5)	52 (83.9)	5 [4,5]
Access to wound care supplies or staff on site to help with wounds	80 (75.5)	42 (52.5)	5 [4,5]
Access to mental health care on site (e.g counselor, therapist)	45 (42.5)	24 (53.3)	5 [4,5]
Availability of harm reduction supplies 24/7 (e.g kits)	66 (62.3)	55 (83.3)	5 [4,5]
Access to sterile syringes	80 (75.5)	55 (68.7)	5 [4,5]
Access to pipes and other materials for safer smoking	61 (57.5)	54 (88.5)	5 [4,5]
Availability of Narcan kits	88 (83.0)	49 (55.7)	5 [5,5]
Area/space dedicated to substance use consumption on or near the site	43 (40.6)	37 (86.0)	5 [4,5]
Smoking area or outdoor space for smoking on site	90 (84.9)	81 (90.0)	5 [4,5]
A common area or community room on site	100 (94.3)	81 (81.0)	5 [3,5]
Lockers on site for your use	79 (74.5)	57 (72.1)	5 [5,5]
Housekeeping of individual rooms by a cleaning crew	87 (82.1)	75 (86.2)	5 [4,5]
Ability to have an outside visitor in your room	6 (5.7)	3(50.0)	5 [3,5]
Daily meals are provided/offered	106 (100)	99 (93.4)	5 [4,5]
Connected to a peer recovery coach on site	29 (27.4)	21 (72.4)	5 [4,5]
Connected to a case manager on site	91 (85.8)	75 (82.4)	5 [4,5]
Events or activities held to support those in recovery / those who wish to get started with recovery, such as support groups on site	38 (35.8)	20 (52.6)	4[3.5,5]
Scheduled community meetings for staff and residents	83 (78.3)	62 (74.7)	4 [2,5]
Provides public transportation support (vouchers, public transportation is reasonable walking distance from site, uber)	57 (53.8)	48 (84.2)	5 [5,5]
*Policies at the harm reduction housing location*			
Staff has Naloxone on person at all times	70 (66.0)	-	5 [5,5]
Metal detectors before entry to site	62 (58.5)	-	4 [3,5]
Security guards on site	104 (98.1)	-	4 [3,5]
Security cameras located throughout the site	96 (90.6)	-	3 [2,5]
Consistent and clear rules or policies related to behavioral misconduct, such as behavioral warnings	94 (88.7)	-	4 [3,5]
27 (32.5)*	-	4 [3,5]	
Absence policy on site (are you able to leave overnight or for extended periods without losing your space?)	100 (94.3)	-	5 [4,5]
Opportunities for paid or unpaid jobs or other activities that residents can be involved with	26 (24.5)	-	4 [3,5]
Room checks / wellness checks conducted by staff	100 (94.3)	-	4 [3,5]
Substance use tolerated in rooms (can you use in your room?)	46 (43.4)	-	4.5 [3,5]

## Data Availability

The datasets used and/or analyzed during the current study are available from the corresponding author on reasonable request.

## Abbreviations

Mass and Cass: Massachusetts Avenue and Melnea Cass Boulevard
HRH: Harm Reduction Housing
PWUD: People Who Use Drugs
HIV: Human Immunodeficiency Virus
PREP: Pre-exposure Prophylaxis
MOUD: Medications for Opioid Use Disorder
EMT: Emergency Medical Technicians
